# Abscisic acid improves drought resilience, growth, physio-biochemical and quality attributes in wheat (*Triticum aestivum* L.) at critical growth stages

**DOI:** 10.1038/s41598-024-71404-4

**Published:** 2024-09-02

**Authors:** Bilal Zulfiqar, Muhammad Aown Sammar Raza, Muhammad Farrukh Saleem, Baber Ali, Muhammad Usman Aslam, Abdullah Ahmed Al-Ghamdi, Mohamed S. Elshikh, Mahmood Ul Hassan, Monika Toleikienė, Junaid Ahmed, Muhammad Rizwan, Rashid Iqbal

**Affiliations:** 1 Institute of Environment and Sustainable Development in Agriculture, Chinese Academy of Agricultural Sciences/Key Laboratory of Agro-Environment, Ministry of Agriculture, Beijing, 100081 People’s Republic of China; 2 Department of Agronomy, Faculty of Agriculture and Environment, The Islamia University of Bahawalpur, Bahawalpur, 63100 Pakistan; 3 Department of Agronomy, University of Agriculture Faisalabad, Faisalabad, Pakistan; 4 School of Science, Western Sydney University, Penrith, 2751 Australia; 5 Department of Botany and Microbiology, College of Science, King Saud University, P.O. 2455, 11451 Riyadh, Saudi Arabia; 6 Department of Ecology and Ecological Engineering, College of Resources and Environmental Sciences, China Agricultural University, 2 W Yuanmingyuan Ave, Haidian, Beijing, 100193 China; 7 Agricultural and Environmental Innovation Research Institute, Liaquatpur, 64000 Pakistan; 8 Institute of Agriculture, Lithuanian Research Centre for Agriculture and Forestry, Instituo Al. 1, 58344 Akademija, Kedainiai, Lithuania; 9 Department of Plant Sciences, Quaid-I-Azam University, Islamabad, 45320 Pakistan; 10 Institute of Crop Science and Resource Conservation (INRES), University of Bonn, 53115 Bonn, Germany; 11 Department of Life Sciences, Western Caspian University, Baku, Azerbaijan

**Keywords:** Abscisic acid, Wheat, Drought, Antioxidants, Water use efficiency, Biochemistry, Plant sciences

## Abstract

Wheat is an important staple crop not only in Pakistan but all over the globe. Although the area dedicated to wheat cultivation expands annually, the quantity of wheat harvested is declining due to various biotic and abiotic factors. Global wheat production and output have suffered as a result of the drought, which is largely driven by a lack of water and environmental factors. Organic fertilizers have been shown to reduce the severity of drought. The current research was conducted in semi-arid climates to mitigate the negative effects of drought on wheat during its critical tillering (DTS), flowering (DFS), and grain filling (DGFS) stages through the application of three different abscisic acid treatments: ABA_0_ (0 mgL^−1^) control, ABA_1_ (100 mgL^−1^) and ABA_2_ (200 mgL^−1^). Wheat growth and yield characteristics were severely harmed by drought stress across all critical development stages, with the DGFS stage being particularly vulnerable and leading to a considerable loss in yield. Plant height was increased by 24.25%, the number of fertile tillers by 25.66%, spike length by 17.24%, the number of spikelets per spike by 16.68%, grain count per spike by 11.98%, thousand-grain weight by 14.34%, grain yield by 26.93% and biological yield by 14.55% when abscisic acid (ABA) was applied instead of the control treatment. Moreover, ABA_2_ increased the more physiological indices (water use efficiency (36.12%), stomatal conductance (44.23%), chlorophyll a (24.5%), chlorophyll b (29.8%), transpiration rate (23.03%), photosynthetic rate (24.84%), electrolyte leakage (− 38.76%) hydrogen peroxide (− 18.09%) superoxide dismutase (15.3%), catalase (20.8%), peroxidase (− 18.09%), and malondialdehyde (− 13.7%)) of drought-stressed wheat as compared to other treatments. In the case of N, P, and K contents in grain were maximally improved with the application of ABA_2_. Through the use of principal component analysis, we were able to correlate our results across scales and provide an explanation for the observed effects of ABA on wheat growth and production under arid conditions. Overall, ABA application at a rate of 200 mgL^−1^ is an effective technique to boost wheat grain output by mitigating the negative effects of drought stress.

## Introduction

Reduced farmland, shifting weather patterns, less predictable precipitation, higher costs for fertilizers and pesticides, and people leaving rural regions in droves are just some of the factors working against agriculture’s expansion. For this reason, new strategies must be used in crop production to boost agricultural output^1,2^. Increasing food production by 50% by 2050 is essential if the globe is to keep up with its fast-expanding population. Rather than increasing the size of the wheat fields, look at different ways to get the highest possible yield^3^. Water shortage is a major problem that has far-reaching consequences for agricultural output. More than half of the world's wheat crop region has also been negatively affected by persistent droughts^4–6^.

One of the most damaging abiotic stresses, drought is responsible for the vast majority of worldwide yield losses^7^. Drought stress is most harmful to wheat at its later phases of growth when it is referred to as terminal drought. Conditions of low atmospheric and soil humidity combined with high air temperature lead to drought because of an imbalance between evaporation and transpiration^8,9^. Tolerant plants have a huge hurdle when trying to strengthen their resistance to oxidative stress by adopting new and better methods^10^. Several factors, such as the spell and timing of the stress, the phenology and genotype of the plant, biotic and abiotic indicators^11,12^, the activation of photosynthesis mechanisms^13,14^ and numerous membrane-bounded transporters as well as respiration activity influence a plant’s response to drought^15,16^.

Stimulating plant growth and development using chemical molecules known as plant growth regulators (PGRs) is a common practice^17,18^. In addition, Kumar et al.^19^ stated that stress-induced PGRs are present, and their control of morphogenesis (root development), meiosis (fruit ripening), oogenesis (embryogenesis), lifespan, water intake, and sensitivity to biotic and abiotic stress have all been linked to stress plants. PGRs such as abscisic acid is also present naturally in plants, although in very small amounts, and can improve and suppress plant growth while allowing for complete regulation of the plant's biochemistry, physiology, and anatomy^20^. ABA, a hormone present in all kingdoms, has been shown to regulate several physiological responses^21^. It controls a wide variety of physiologic processes in vascular plants, such as dormancy of seeds^19^, cambium activity^22^, organ genesis^23^, and fruit ripening^24^. However, it has a role in controlling the physiological reactions to environmental stresses such as heat, salt, and dehydration^25–27^.

There are a wide variety of environmental factors, including drought, salt, and cold, that may naturally threaten that plant's response by producing the hormone abscisic acid (ABA), demonstrating its high agronomic potential^28^. Drought was considered an interesting topic of research, with the processes underlying ABA activity on both the molecular and physiological levels and their findings have already been summarised^29–31^. Exogenous ABA has been found to decrease reactive oxygen species (ROS), however, the evidence supporting this concept is still mixed. Many plant species have reported higher endogenous ABA levels in response to drought stress^32^. Build-up via upregulating the activity or expression levels of several antioxidative enzymes^33^. For instance, in plants of the kiwifruit family that are under stress due to drought, exogenous ABA treatment significantly increases peroxidase (POD), catalase (CAT), and superoxide dismutase (SOD)^34^. Cotinus, while under drought stress, has a decreased concentration of ABA increase in CAT activity, although it has a dramatic effect on SOD and POD activity in this species^35,36^.

Furthermore, the external treatment of ABA during periods of water stress expedited the buildup of osmolytes. It enhanced the hydration level of grains, leading to increased grain weight in vulnerable wheat cultivars^37^. In addition, the application of ABA to pre-soak seeds resulted in a considerable increase in the activity of antioxidant enzymes, including SOD, POD, CAT, ascorbate peroxidase (APX), and glutathione reductase (GR), in maize seedlings^38^. Similarly, it was shown that the plants treated with ABA had a greater relative water content (RWC) compared to the control plants when exposed to drought stress. The number^38^. Travaglia et al.^39,40^ found that applying ABA directly to the leaves of wheat during the reproductive stage effectively reduced the negative impact of drought. Research has shown that ABA is a crucial ingredient in mitigating oxidative damage by stimulating the antioxidant defence system in maize seedlings experiencing water restriction^41,42^.

Numerous research studies have highlighted the positive impact of abscisic acid on promoting wheat growth. Nonetheless, information is scarce regarding the enzymatic activities and other physiological mechanisms of wheat in response to drought stress at different growth stages of wheat. Therefore, this novel study was performed with the following aims: the influence of ABA on growth and physiological indicators during critical growth stages of wheat under drought circumstances, the efficacy of ABA through foliar spray, and assess it as a potential approach to mitigate the negative consequences of drought on the crucial growth stages (tillering, flowering, and grain-filling) of wheat. We forecast that the foliar application of ABA will boost wheat's growth, water-related metrics, physiological characteristics, and productivity in dry environments.

## Materials and methods

### Experimental layout and crop husbandry

This pot experiment was conducted at the experimental station of The Islamia University of Bahawalpur, Pakistan (29° 23′ 60.00′′ N, 71° 40′ 59.99′′ E) with a factorial arrangement of the complete randomized design (CRD). There were 3 treatments of different concentration of foliar application of ABA (ABA_0_ = 0 mgL^−1^, ABA_1_ = 100 mgL^−1^, and ABA_2_ = 200 mgL^−1^) was applied on each development stages (tillering (DTS), flowering (DFS), and grain filling (DGFS) phases). While, drought was enforced on specific one stage in each pot according to the treatment with three replications. Furthermore, full irrigation was used as a control (CK) and plants in the control treatment were sprayed with the same amount of deionized water^43^.

In this experiment, used Galaxy 2013 wheat cultivar selected after the screening of 5 different verities such as Punjab 2011, Faisalabad 2008, Lasani 2008, 8200 and Galaxy 2013. Moreover, this verity is also recommended in this semi-arid region by Regional Agricultural Research Institute (RARI) Bahawalpur ^44^. On November 10th, 2019, wheat seeds were sown in the 36 pots (26 × 29 cm; three for each application) filled with six kg of soil and seeds were purchased from RARI Bahawalpur. Fertilizers 0.52 g N, 0.35 g P, and 0.23 g K were added in each pot according to the recommended dose of N, P, and K (160–115–75 kg/ha) in wheat. The soils physiochemical composition is shown in Table 1. In the event of inclement weather, a transparent plastic cover was put over the wire enclosure to shield the plants within. Up to the point of complete emergence, all containers were watered uniformly. After 20 days of seeding, a total of four plants were kept in each container by removing the excess. A translucent plastic canopy was placed over the wire-house to protect the crop from rainfall when needed. All the containers were watered equally until the plants fully emerged.

**Table 1:** Physicochemical characteristics of the soil.

Parameters	Contents
Sand	53.5%
Clay	14.5%
Silt	32%
Ph	7.36
Texture	Sandy-loam soil
Ammoniac N (mg g^−1^)	1.48
Electric conductivity (dSm^−1^)	2.63
Available potassium (ppm)	108
Available phosphorus (ppm)	6.23
Organic matter (%)	0.88

### ABA application and drought imposition

In this experiment used three different concentrations (ABA_0_ = 0 mgL^−1^, ABA_1_ = 100 mgL^−1^, and ABA_2_ = 200 mgL^−1^) of foliar ABA during three DFS, DTS, and DGFS observed development stages of wheat. Since the drought started, every pot has been watered in the same manner. Then, throughout the flowering (DFS), tillering (DTS), and grain filling (DGFS) phases, under drought we kept the water holding capacity (WHC) of each pot at 35% during 40–50 days after sowing (DAS) for DTS, 80–90 DAS for DFS, and 100–110 DAS for DGFS, whereas the control was kept at 85% WHC using tensiometer^44–47^.

### Growth and yield parameters

The growth, yield, and various attributes of wheat were measured, including spike length (cm), number of grains per spike, number of spikelets per spike, number of fertile tillers, 1000-grain weight (g), plant height (cm), organic yield per plant (g), grain yield per plant (g), and harvest index. These measurements were obtained using established procedures and protocols. The yield of wheat plants was obtained by physically harvesting them at the end of their life cycle. Similarly, to measure the height of the plants, three plants were randomly chosen from each pot and their height was measured from the surface of the soil to the spikelet using a ruler calibrated in millimetres and centimetres. To measure the length of the spike and the number of grains per spike, use a centimetre scale to measure the distance from the base to the terminal spikelet of three randomly chosen plants. Additionally, count the number of grains in ten randomly selected clusters of seeds. In addition, the digital balance was used to measure the 1000-grain weight, taking advantage of its precision of 0.01 g. The following formula was used to determine the harvest index (HI):

$$HI=\frac{\text{Grain Yield}}{\text{Biological Yield}} \times 100$$

### Determination of physiological parameters

In accordance with the method employed by Khalilzadeh et al.^48^, 0.5 g of recently harvested leaf tissue was slowly mixed with 10 mL of acetone (80%) to determine the chlorophyll levels. The mixture was then subjected to centrifugation at a speed of 400 rpm for a duration of 10 min. The spectrophotometer recorded the absorbance at wavelengths of 645 nm, 663 nm, and 470 nm. The chlorophyll contents were acquired using the following method:

$$ {\text{Chlorophyll}}\,{\text{a}} = \left( {{19}.{3}0 \, \times {\text{ A663}} - 0.{86} \times {\text{A645}}} \right) \times \left( {{\text{V /1}}00{\text{ W}}} \right) $$

$$ {\text{Chlorophyll}}\,{\text{b}} = \left( {{19}.{3}0 \times {\text{A645}} - {3}.{6} \times {\text{A663}}} \right) \times \left( {{\text{V/1}}00{\text{ W}}} \right) $$

Raza et al.^47^ defined a mathematical equation for determining the water usage efficiency (WUE, g pot^−1^ mm^−1^) as follows:

$$ {\text{WUE}} = {\text{Grain}}\,{\text{Yield}}/{\text{Total}}\,{\text{Water}}\,{\text{Applied}} $$

Automatic porometer MK-3 Delta-T Devices, BC, England, was used to measure the transpiration rate and stomatal conductance (SC). It was measured from 5 leaves per pot between 11:00 and 13:00 h, when atomosphre was cleared and stomata were fully opened and photosynthetic activity were high^49^.

### Determination of electrolyte leakage and hydrogen peroxide concentration

The shoot samples were vertically inserted into tubes to measure electrolyte leakage percentage and heated in distilled water at 32 °C for 2 h. After this step, the electrical conductivity (EC) of the solution was determined as EC1. Subsequently, the solution was heated at a constant temperature of 121 °C for 20 min. EL was calculated using a previously defined equation ^50^.

$$EL=\frac{EC1}{EC2} \times 100$$

To determine the concentration of hydrogen peroxide (H_2_O_2_), a 50 mg leaf tissue was mixed with 3.0 mL of phosphate buffer solution and centrifuged at 6000*g* for 30 min at 4 °C. Then an upper layer of the centrifuges sample was mixed with 1.0 mL of (0.1%) titanium sulphate by centrifuging at 6000*g* for 20 min at 4 °C. Specifically, the absorbance was measured at 410 nm. The extinction coefficient for H_2_O_2_ was found to be 0.28 mol^−1^ cmss^−1^.

### Estimation of antioxidant enzymes

The activity of superoxide dismutase (SOD) was measured using the procedure outlined by Giannopolitis and Ries^51^. 2 µM riboflavin was added to a 3 mL reaction mixture containing 50 mM phosphate buffer (pH 7.8), 13 mM methionine, 75 µM nitro blue tetrazolium, 0.1 µM EDTA, and 0–100 µl of enzyme extract^51^. The tubes were shaken and lighted with a 15-Watt fluorescent bulb. The reaction was allowed to proceed for a duration of 10 min. Subsequently, the light source was turned off and the absorbance was measured at a wavelength of 560 nm. A single unit of SOD activity was determined as the quantity of enzyme needed to induce a 50% decrease in the rate of nitroblue tetrazolium chloride reduction.

An approach for determining CAT activity was suggested by Hwang et al.^52^, which involves monitoring the rate of H_2_O_2_ breakdown at 240 nm. Modifications were made to the guaiacol oxidation procedure reported by Maechlay and Chance^53^ to determine POD activity. The reaction mix included 50 mM potassium phosphate buffer (pH 6.1), 1% guaiacol, 0.4% H_2_O_2_, and extracted enzyme, and it took up a total volume of 3 ml. At 470 nm, an increase in absorbance (E = 25.5 mM^−1^ cm^−1^) was seen owing to oxidation of guaiacol^54^. MDA contents were estimated as described by Wu et al.^55^.

### Grain quality parameters

The levels of nitrogen (N), phosphorus (P), and potassium (K) were examined to evaluate the quality of the grain. The estimation of total nitrogen from wheat seeds was conducted using the Micro Kjeldahl's technique, as described by Piper^56^. To assess the P content in wheat seed, the vanado-phosphate molybdate yellow colour technique proposed by Kitson and Mellon^57^ was used on a di-acid extract. To assess the K level in wheat seed, a flame photometer with a di-acid extract method, as reported by Piper^56^, was used.

### Statistical analysis

**Fig. 1:**
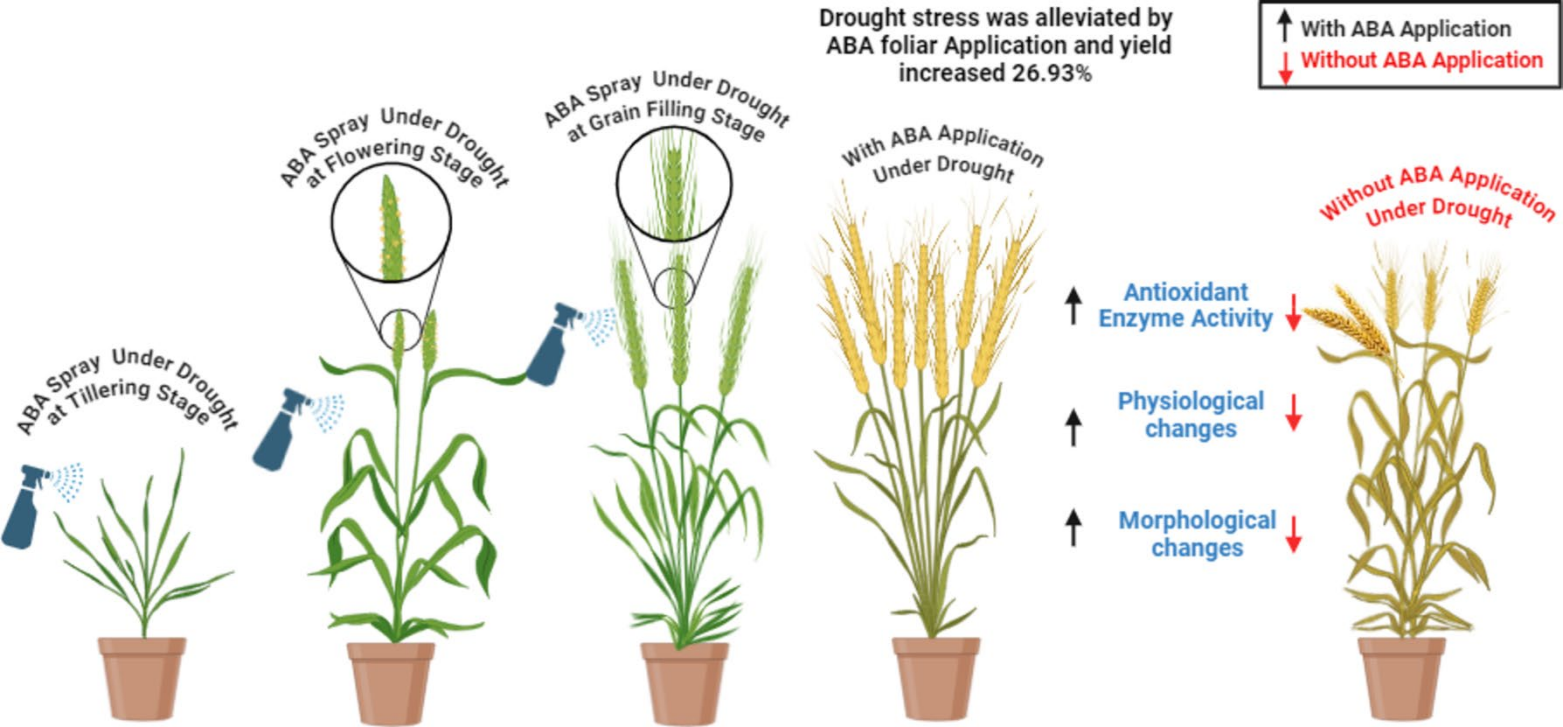
Graphical abstract about role of foliar application of Abscisic Acid under drought stress at different growth stages of wheat.

The analysis of variance (ANOVA) was calculated using STATISTIX software (version 8.1) on the present data with the least significant difference (Tukey’s HSD) at the 5% probability level to compare the means of the variables^58^. A biplot graph was created in Origin Pro 9.1 to visualize the principal component analysis (PCA) findings (Fig. 1).

## Results

### Plant height and spike length

The imposition of drought stress caused a significant reduction in plant height by 34.9%, 32.1%, and 24.3%, and spike length by 38.6%, 33.1%, and 26.6% at the DTS, DFS, and DGFS, respectively as compared to CK (no drought at any stage). However, the ABA applications (ABA_1_ and ABA_2_) mitigated the impact of drought stress, leading to an increase in plant height and spike length by 18.7%, 24.2%, and 13.5%, 17.2%, respectively as compared to control treatment ABA_0_ (Table 2).

**Table 2:** The effect of abscisic acid on plant height (PH, cm), spike length (SL, cm), number of spikelets per spike (NSPS), number of grains per spike (NGPS), number of fertile tiller (NFT) of wheat under different drought episodes.

Treatments	PH	SL	NSPS	NGPS	NFT
CK (Control, No Drought at any Stage)					
ABA_0_ (0 mgL^−1^)	52.23 ± 1.88 b	9.50 ± 0.81 abc	14.13 ± 0.97 abc	35.13 ± 0.97 ab	6.73 ± 0.33 abc
ABA_1_ (100 mgL^−1^)	58.10 ± 1.09 a	10.65 ± 0.73 ab	15.34 ± 1.15ab	37.00 ± 0.94 a	7.53 ± 0.39 ab
ABA_2_ (200 mgL^−1^)	61.23 ± 1.02 a	11.13 ± 0.64 a	16.62 ± 0.86 a	39.23 ± 1.07 a	8.10 ± 0.33 a
DTS (Drought at tillering)					
ABA_0_ (0 mgL^−1^)	36.53 ± 0.97 ef	6.93 ± 0.97 c	11.43 ± 0.94 abc	28.83 ± 0.86 cde	5.03 ± 0.34 cde
ABA_1_ (100 mgL^−1^)	43.01 ± 0.95 cd	7.71 ± 0.70 bc	12.81 ± 0.95 abc	30.81 ± 0.95 bcde	5.91 ± 0.38 abcde
ABA_2_ (200 mgL^−1^)	47.62 ± 1.03 bc	7.92 ± 0.65 bc	14.22 ± 1.03 abc	34.02 ± 1.03 abc	7.02 ± 0.45 abc
DFS (Drought at flowering)					
ABA_0_ (0 mgL^−1^)	32.40 ± 1.14 f.	6.80 ± 0.63 c	10.40 ± 1.05 bc	27.20 ± 1.05 de	4.41 ± 0.48 de
ABA_1_ (100 mgL^−1^)	47.30 ± 1.05 bc	8.00 ± 0.49 bc	11.00 ± 0.97 bc	29.30 ± 0.98 cde	5.44 ± 0.45 bcde
ABA_2_ (200 mgL^−1^)	50.10 ± 1.35 b	8.70 ± 0.82 abc	12.30 ± 0.91 abc	31.10 ± 0.88 bcd	6.12 ± 0.40 abcd
DGFS (Drought at grain filling)					
ABA_0_	38.40 ± 1.06 de	7.10 ± 0.62 c	9.40 ± 0.85 c	25.60 ± 0.99 e	3.80 ± 0.46 e
ABA_1_	47.90 ± 0.72 bc	8.70 ± 0.64 abc	9.80 ± 0.98 c	26.70 ± 1.00 de	4.83 ± 0.43 cde
ABA_2_	51.70 ± 0.66	8.90 ± 0.72 abc	11.30 ± 0.95 bc	28.30 ± 1.01 de	5.60 ± 0.49 bcde
HSD (*p* < 0.05)	**5.33**	**3.04**	**5.25**	**5.33**	**2.29**
Significance					
Drought	***	**	***	***	**
ABA	***	**NS**	**	***	***
Drought × ABA	**	**NS**	**NS**	**NS**	***

ABA_0_, ABA_1_ and ABA_2_ treatments indicates 0 mgL^−1^ (control), 100 mgL^−1^, and 200 mgL^−1^ of ABA respectively. Ck, DTS, DFS, and DGFS treatments indicates no drought at any stage, drought at tillering, drought at flowering and grain filling stages respectively. There were no significant differences at the 5% probability level among the means that share the same letter case. NS = non-significant, * = significant at *p* ≤ 0.05, ** = significant at *p* ≤ 0.01 and *** = significant at *p* ≤ 0.001.

Significant values are in bold.

### Yield attributes

The drought conditions at specific growth stages (DTS, DFS, and DGFS) lowered the spikelet’s numbers per spike (19.8, 36.7, and 51.1%), the number of fertile tillers (24.6, 40.5, and 57.4%), the grain number per spike (18.8, 27.1, and 38.1%), the 1000-grain weight (12.7, 25.4, and 35.5%), the grain yield (13.0, 31.1, and 40.4%), the biological yield (17.1, 39.0, and 76.5%), and the harvest index (3.3, 4.9, and 20.6%) as compared to control (CK) when no drought imposed at any stages. Under both drought and control conditions, ABA demonstrated a positive effect, mitigating the adverse effects of drought and enhancing the values of the aforementioned parameters. Notably, a foliar application of ABA_**2**_ at 200 mgL^−1^ resulted in a significantly higher grain yield (26.9%) compared to the application of ABA_**1**_ at 100 mgL^−1^ and ABA_**0**_ control treatment (Table 3). Moreover, ABA foliar treatment effectively reduced the negative impact of drought and simultaneously enhanced the potential of various yield-related characteristics of wheat, including NSPS, NFT, NGPS, 1000Gwt, GY, BY, and HI (Tables 2, 3).

**Table 3:** The effect of ABA on 1000-grain weight (1000 Gwt, g), grain yield per plant (GY, g), biological yield per plant (BY, g), harvesting index (HI, %) of wheat under different drought episodes.

Treatments	1000 Gwt	GY	BY	HI
CK (Control, No Drought at any Stage)				
ABA_0_	30.23 ± 0.97 abc	6.67 ± 0.48 ab	15.20 ± 0.80 a	42.73 ± 1.14 f.
ABA_1_	32.41 ± 0.95 ab	7.73 ± 0.45 a	15.43 ± 0.92 a	50.51 ± 1.21 de
ABA_2_	34.87 ± 0.78 a	8.23 ± 0.50 a	16.20 ± 0.97 a	51.08 ± 1.23 de
DTS (Drought at tillering)				
ABA_0_	27.00 ± 0.89 bcd	5.73 ± 0.39 abc	12.33 ± 0.89 abcd	46.52 ± 1.04 ef
ABA_1_	29.32 ± 0.96 bc	6.83 ± 0.57 ab	13.43 ± 0.92 abc	50.98 ± 1.46 de
ABA_2_	30.12 ± 1.03 abc	7.48 ± 0.54 a	14.40 ± 0.91 ab	51.99 ± 1.29 cd
DFS (Drought at flowering)				
ABA_0_	23.40 ± 1.06 cd	4.31 ± 0.29 bc	9.70 ± 0.57 abcd	44.25 ± 1.21 f.
ABA_1_	26.70 ± 1.00 cd	6.10 ± 0.48 abc	11.70 ± 0.76 abcd	52.12 ± 1.09 cd
ABA_2_	27.53 ± 1.02 cd	6.83 ± 0.53 ab	12.30 ± 0.90 abcd	55.64 ± 1.32 c
DGFS (Drought at grain filling)				
ABA_0_	21.34 ± 0.96 e	4.13 ± 0.42 c	7.70 ± 0.98 cd	54.04 ± 1.17 cd
ABA_1_	24.30 ± 0.80 de	5.73 ± 0.49 abc	8.81 ± 0.92 d	65.77 ± 1.24 a
ABA_2_	26.50 ± 0.95 de	6.07 ± 0.53 abc	10.13 ± 0.75 d	59.93 ± 1.61 b
HSD (*p* < 0.05)	**4.99**	**2.57**	**4.64**	**4.23**
Significance				
Drought	**	***	***	***
ABA	***	***	**	***
Drought × ABA	***	**NS**	**NS**	**

ABA_0_, ABA_1_ and ABA_2_ treatments indicates 0 mgL^−1^ (control), 100 mgL^−1^, and 200 mgL^−1^ of ABA respectively. Ck, DTS, DFS, and DGFS treatments indicates no drought at any stage, drought at tillering, drought at flowering and grain filling stages respectively. There were no significant differences at the 5% probability level among the means that share the same letter case. NS = non-significant, * = significant at *p* ≤ 0.05, ** = significant at *p* ≤ 0.01 and *** = significant at *p* ≤ 0.001.

Significant values are in bold.

### Chlorophyll concentrations

**Fig. 2:**
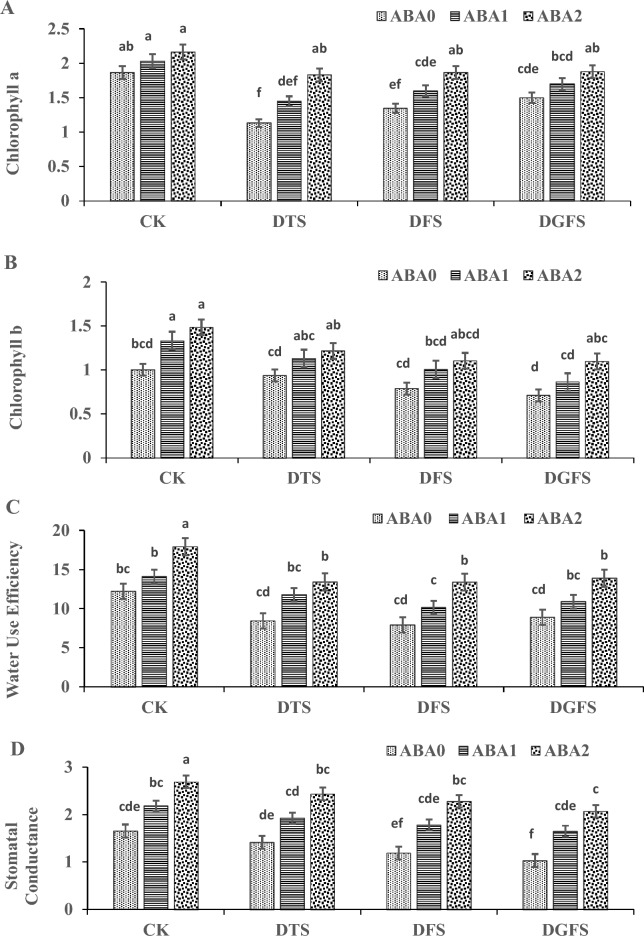
Illustrates the impact of ABA application on stomatal conductance (m mol^−2^ s^−1^), water use efficiency (%), and Chlorophyll a and b (mg g^−1^ FW) contents at critical growth stages of wheat under drought stress. The labels CK, DGFS, DFS, and DTS represent the control as no drought at any stage, drought at grain filling, flowering, and tillering stages, respectively. ABA_0_, ABA_1_ and ABA_2_ indicates control, 100mgL^−1^ and 200 mgL^−1^ ABA application respectively. The error bars in the figure indicate the standard error (n = 3).

The application of ABA had a significant impact on the chlorophyll content (a and b) of wheat leaves under drought as shown in Fig. 2. Compared to the control (ABA_0_) with 100 and 200 mgL^−1^ concentrations of ABA (ABA_1_ and ABA_2_) significantly enhanced the chlorophyll content a and b by 14.0%, 25.7%, and 10.9%, 23.1%, respectively. Moreover, drought stress led to reductions in Chlorophyll a and b levels by 37.3%, 26.0%, and 19.4%, and 16.3%, 31.9%, and 43.0%, respectively, at DTS, DFS, and DGFS stages of wheat as compared to CK when no drought was imposed at any growth stages. Notably, under drought, the application of ABA_2_ resulted in more pronounced effects on chlorophyll content.

### Water use efficiency and stomatal conductance

ABA applications (ABA_1_ and ABA_2_) significantly reduced the drought impact by 29.8% and 44.2%, respectively, as compared to the control (ABA_0_). The application of ABA at different development stages resulted in increased efficiency of wheat water consumption under drought (Fig. 2). Water use efficiency (WUE) decreased by 31.1%, 40.8%, and 32.1% under drought at DTS, DFS, and DGFS, respectively, in comparison to the control (CK) when no drought was imposed at any stages. However, the application of ABA (ABA_1_ and ABA_2_) significantly mitigated the impact of drought, reducing the WUE decline by 20.2% and 36.1%, respectively, compared to the non-ABA treated plants. Additionally, stomatal conductance (m mol m^−2^ s^−1^) was reduced by 13.0%, 24.3%, and 37.4% under drought stress at DTS, DFS, and DGFS, respectively, in comparison to the control treatment (CK). Nevertheless, ABA_1_ and ABA_2_ applications significantly alleviated the drought impact, reducing the stomatal conductance decline by 29.8% and 44.2%, respectively, compared to the control treatment ABA_0_.

### Gas exchange attributes

**Fig. 3:**
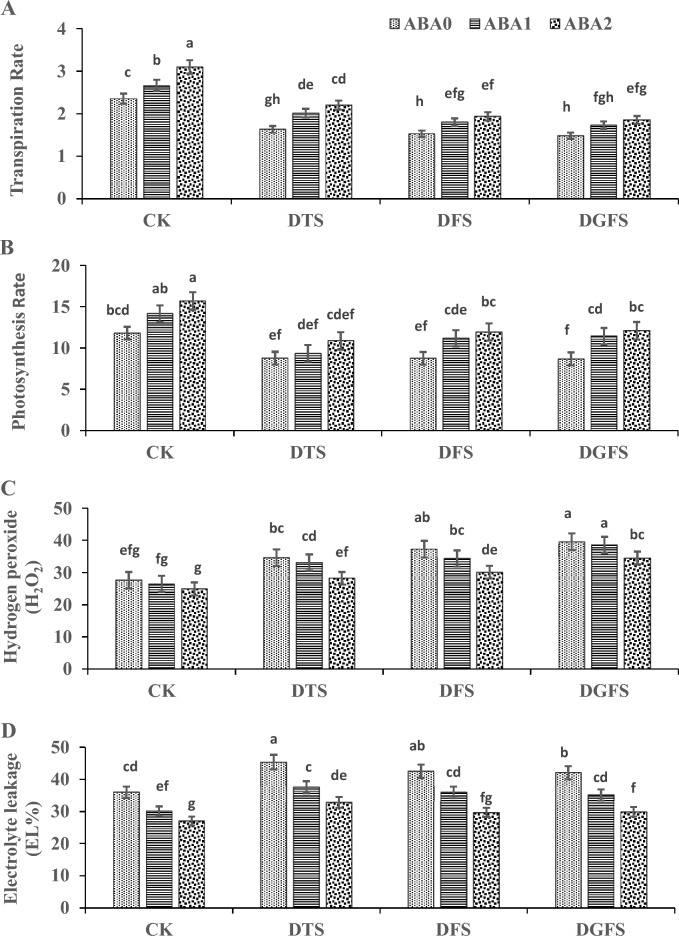
Illustrates the impact of ABA application on Photosynthetic rate (μmol CO_2_ m^−2^ S^−1^), transpiration rate (mmol H_2_O m^−2^ S^−1^), Hydrogen peroxide (μM g^−1^ FW) and Electrolyte leakage (%) under drought stress at critical growth stages of wheat. The labels CK, DGFS, DFS, and DTS represent the control as no drought at any stage, drought at grain filling, flowering, and tillering stages, respectively. ABA_0_, ABA_1_ and ABA_2_ indicates control, 100mgL^-1^ and 200mgL^-1^ ABA application respectively. The error bars in the figure indicate the standard error (n = 3).

Drought stress caused a significant reduction in transpiration (38.9%, 54.3%, and 60.2%) and photosynthesis activities (43.7%, 30.8%, and 29.4%) at DTS, DFS, and DGFS, respectively, compared to the control (CK) when no drought was imposed at any stage. ABA_1_ and ABA_2_ applications ominously increased transpiration and photosynthesis by 14.7%, 23.0%, and 17.5%, 24.9%, respectively, in comparison to the control ABA_0_ treatment when no application of ABA under drought (Fig. 3A,B).

### Antioxidant enzymatic activities

Under drought stress, H_2_O_2_ and EL percentage were elevated by 17.6%, 22.4%, and 29.8% for H_2_O_2_ and 19.5%, 13.8%, and 12.9% for EL, at the DTS, DFS, and DGFS stages of wheat, respectively, as compared to control when no drought was imposed at any stage (Fig. 3C,D). However, the application of ABA_1_ and ABA_2_ (Fig. 3C,D) significantly reduced the levels of H_2_O_2_ (− 4.9% and − 18.0%) and EL (− 19.5% and − 38.7%) in the leaves under drought stress as compared to control treatment (ABA_0_).

**Fig. 4:**
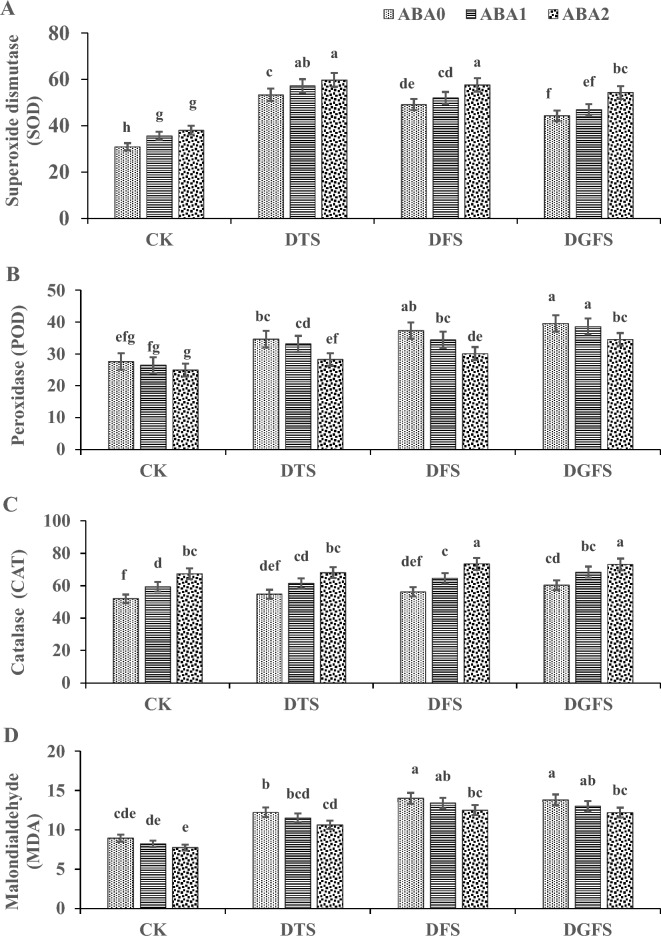
Illustrates the impact of ABA application on Superoxide dismutase (μmol min^−1^ mg^−1^ protein), Peroxidase (μmol min^−1^ mg^−1^ protein), Catalase (μmol min^−1^ mg^−1^ protein) and Malondialdehyde (μmol min^−1^ mg^−1^ protein) activities under drought stress at critical growth stages of wheat. The labels CK, DTS, DFS, and DGFS represent the control as no drought at any stage, drought at tillering, flowering, and grain-filling stages, respectively. ABA_0_, ABA_1_ and ABA_2_ indicates control, 100 mgL^−1^ and 200 mgL^−1^ ABA application respectively. The error bars in the figure indicate the standard error (n = 3).

The application of ABA under drought conditions had a significant impact on the antioxidant enzymatic activities of wheat leaves, as demonstrated in Fig. 4. Drought stress influence the enzymatic activities of SOD, CAT, POD, and MDA as compare to control treatments (CK and ABA_0_). However, the addition of ABA_1_ and ABA_2_ during the DTS, DFS, and DGFS stages substantially modulated these activities when compared with ABA_0_. SOD and CAT activities (Fig. 4A,C) were decreased by 38.5%, 34.0%, 28.0%, and 3.1%, 8.1%, 11.4%, respectively, when compared to CK, no drought was imposed. Moreover, ABA_1_ and ABA_2_ applications significantly increased SOD and CAT activities by 7.3%, 15.3%, and 12.0%, 20.8%, respectively, in comparison to the control (ABA_0_) under drought conditions.

While, drought at DTS, DFS, and DGFS boosted the POD and MDA levels by 17.6%, 22.4%, 29.8%, and 27.6%, 37.6%, 36.2%, respectively, as compared to CK. But, application of ABA_1_ and ABA_2_ significantly diminished the POD and MDA activities under drought by − 4.9%, − 18.0%, and − 6.1%, − 13.7%, respectively, in comparison to the control treatment ABA_0_ (Fig. 4B,D). Furthermore, ABA_2_ (200 mgL^−1^) application was more significant results than ABA_1_ (100 mgL^−1^) under drought, for antioxidant enzymatic activities.

### Nutrient percentage in grains

**Fig. 5:**
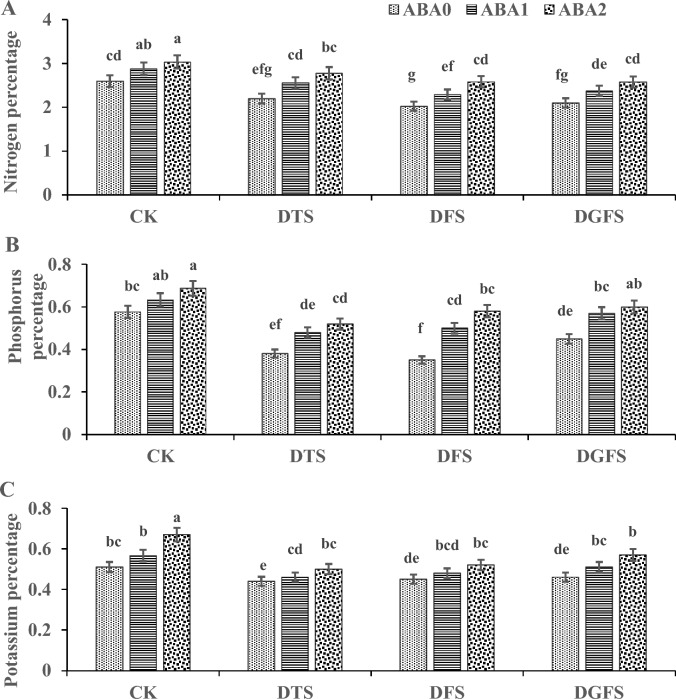
Illustrates the impact of ABA application on Nitrogen (%), Phosphorus (%), and Potassium (%) affected under drought stress at critical growth stages of wheat. The labels CK, DTS, DFS, and DGFS represent the control as no drought at any stage, drought at tillering, flowering, and grain filling stages, respectively. ABA_0_, ABA_1_ and ABA_2_ indicates control, 100 mgL^−1^and 200 mgL^−1^ABA application respectively. The error bars in the figure indicate the standard error (n = 3).

The results regarding the percentage of N, P, and K in wheat grains enhanced with the application of ABA under drought episodes are shown in Fig. 5A–C. Drought stress at critical growth stages (DTS, DFS, and DGFS) reduced the percentage of nitrogen (− 12.9, − 23.2, and − 20.7%), phosphorus (− 37.4, − 32.6, and − 17.1%), and potassium (− 24.7, − 20.6, and − 13.4%) respectively, as compared to CK. Moreover, ABA_1_ and ABA_2_ significantly enhanced the N (11.6% and 18.7%), P (19.5% and 26.3%), and K (7.7% and 17.6%) respectively when compared with no application of ABA_0_ under drought.

### Principal component analysis

**Fig. 6:**
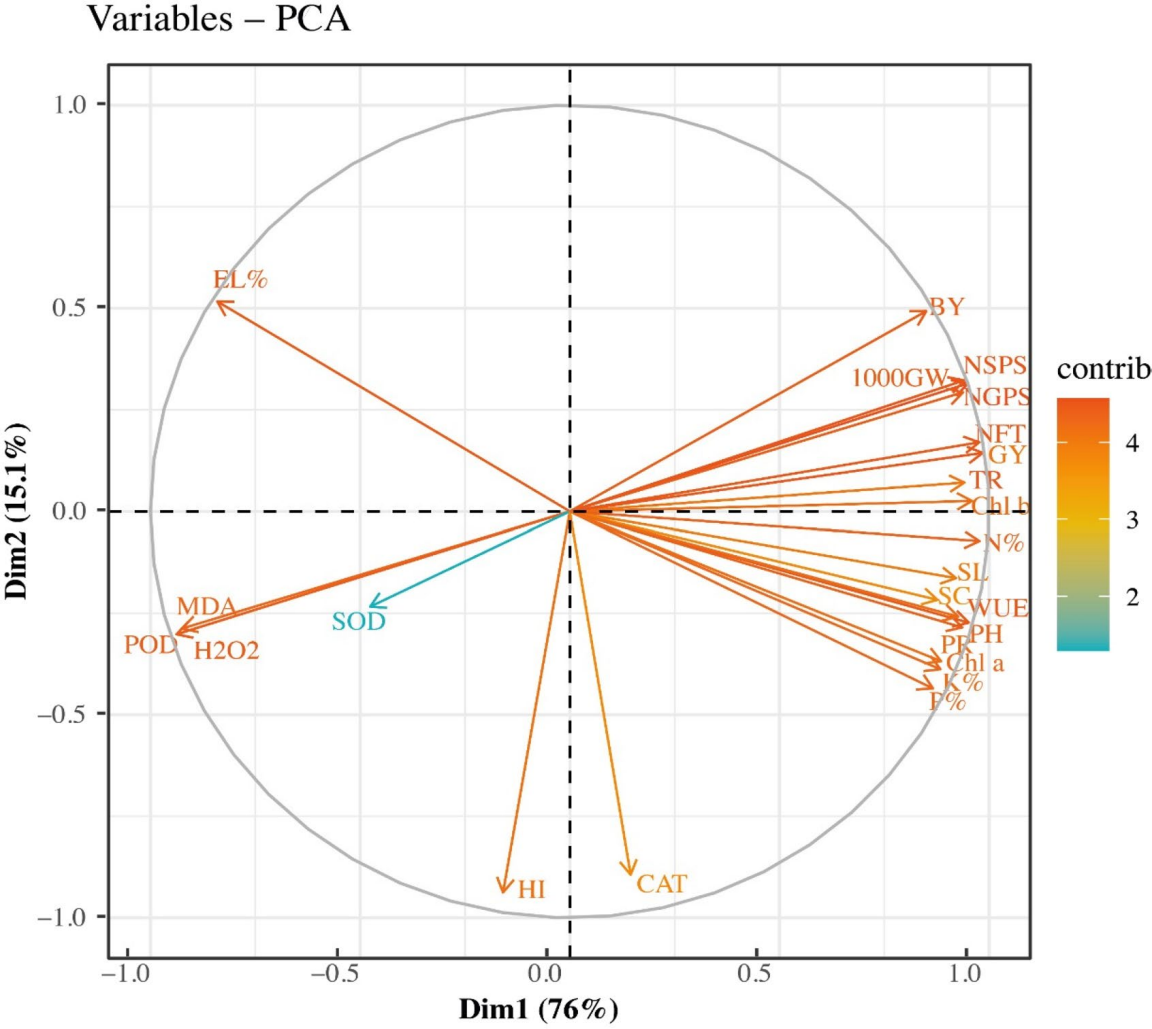
Principal component analysis among the recorded variables during this study. Plant height (PH, cm), spike length (SL, cm), number of grains per spike (NGPS), number of spikelets per spike (NSPS), number of fertile tillers (NFT), 1000 grain weight (GW, g), grain yield per plant (GY, g), biological yield per plant (BY, g), harvesting index (HI), chlorophyll a (Chl a), chlorophyll b (Chl b), photosynthesis rate (PR), transpiration rate (Tr), water use efficiency (WUE), stomatal conductance (SC), hydrogen peroxide (H2O2), malondialdehyde (MDA), superoxide dismutase (SOD), peroxidase (POD), electro leakage percentage (EL%), nitrogen (N%), phosphorus (P%), and potassium (K%).

**Fig. 7:**
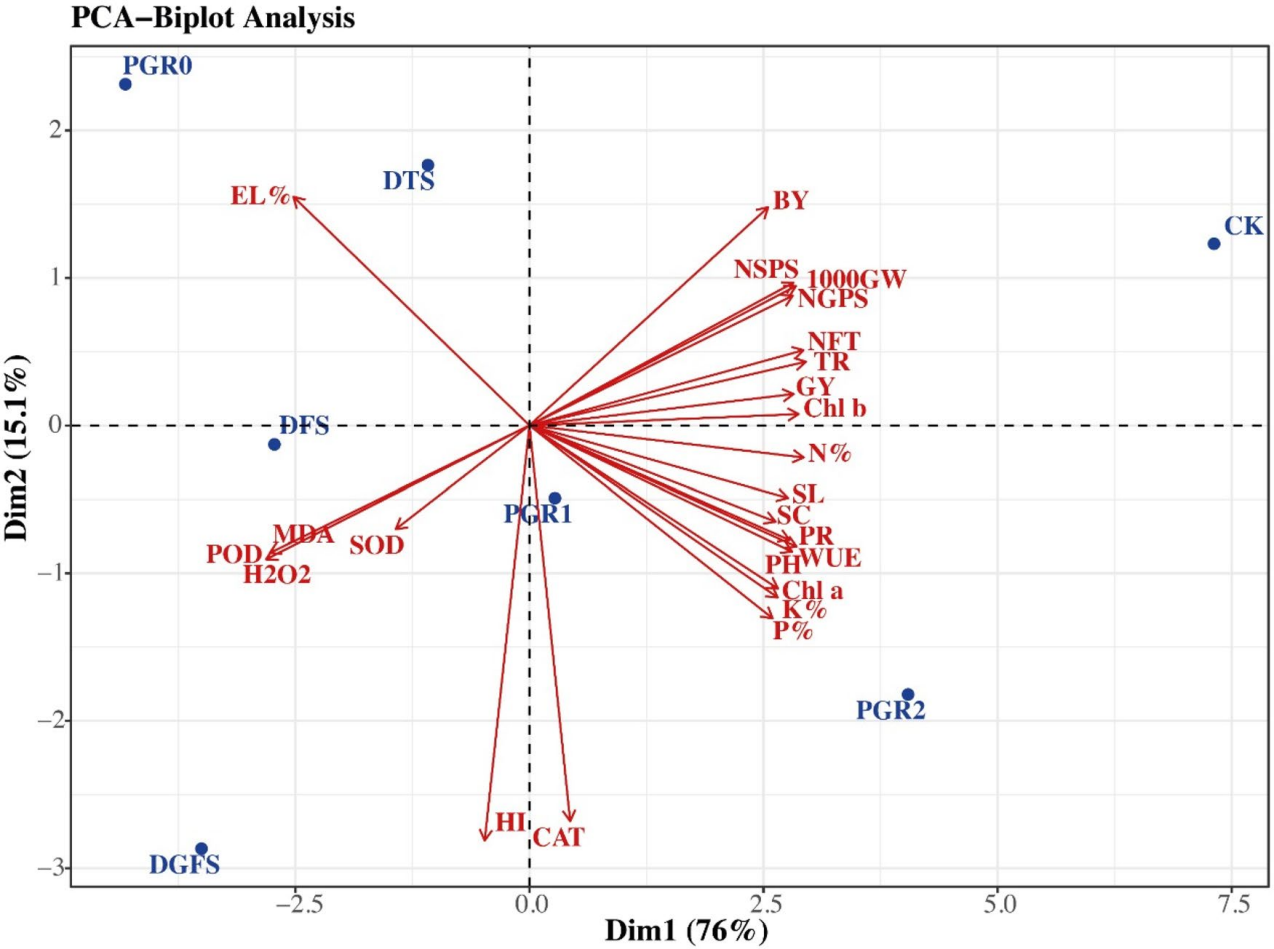
Principal component analysis PCA-biplot among the recorded variables and treatments during this study. Plant height (PH, cm), spike length (SL, cm), number of grains per spike (NGPS), number of spikelets per spike (NSPS), number of fertile tillers (NFT), 1000 grain weight (1000 GW, g), grain yield per pot (GY, g), biological yield per pot (BY, g), Harvesting Index (HI), chlorophyll a (Chl a), chlorophyll b (Chl b), photosynthesis rate (PR), transpiration rate (Tr), water use efficiency (WUE), stomatal conductance (SC), hydrogen peroxide (H_2_O_2_), malondialdehyde (MDA), superoxide dismutase (SOD), peroxidase (POD), electro leakage percentage (EL%), nitrogen (N%), phosphorus (P%), and potassium (K%). Application of ABA 0 mg/L (PGR0), ABA 100 mg/L (PGR1), and ABA 200 mg/L (PGR2) under drought stress condition at different growth stages Control (CK), Drought at tillering stage (DTS), drought at flowering stage (DFS), and drought at grain filling stage (DGFS).

Principal component analysis (PCA) has been widely employed to analyze the variations and associations among different growth, physiological, morphological, biochemical, yield, and quality-linked attributes of wheat under the application of nutrient^44,59^. In this study, we also conducted PCA to evaluate the variability and associations among agronomic traits and the distribution of treatments (see Figs. 6 and 7). In Fig. 6, agronomic traits which are denoted with different vectors were used and spread among the different clades. In Fig. 7, both agronomic traits and treatments were used and a biplot was created. The cumulative variance of the first two PCs was 91.1% of the total variance (Fig. 6, 7). The first two PCs (PC1 = 76%, and PC2 = 15.1%) were significant. All the studied parameters were the major contributing factors in PC1 except EL%, MDA, POD, SOD, H_2_O_2_, HI, and CAT. Similarly, in PC2, SOD was the main factor and showed the highest loading values (Fig. 6). Projection of traits showed that MDA, H_2_O_2_, and POD were strongly related to each other. Similarly, in the PCA-biplot, the treatments scattered away from each other and indicated clear differences among them (Fig. 7). Based on PCA results, these parameters can be utilized in future breeding programs.

## Discussion

The main goal of this study was to identify the most effective doses of ABA for foliar application, aiming to expedite wheat growth and alleviate the adverse impacts of drought (Fig. 1). Notably, substantial improvements in growth attributes were observed when applying a dosage of ABA 200 mgL^−1^ under stressful conditions. Reduced wheat harvests may be attributed to drought stress, a global growth limitation^60^. Low water availability might stunt a plants growth^61^. Wheat plant development is drastically altered by ABA treatments. Hussain et al.^62^ found that water stress significantly affected cell division. The disruption of protoplasmic processes caused by diminished turgidity and dehydration leads to less cell division and shorter plants as a result. Plant growth regulators are very useful here since they increase plant height. Drought stress, at any stage of the development cycle, reduced the plants height^63^. Possible hormonal changes during drought stress might have a significant impact on plant height^64^. Our result also in line with the previous researches and showed that the height of wheat was increased by ABA_1_ and ABA_2_ application as compared to the control under stress.

Plants with longer spikes are more likely to produce a greater number of spikelets and a better production^44^. Lack of water in many plant functions, as described by Fahad et al.^65^ reduces the number of spike lengths. According to Iqbal^66^, using ABA improve the nutritional availability, which in turn results in longer spikes. Specifically, proline synthesis is increased by ABA under dry circumstances, which in turn increases spike length and enhances plant metabolic processes^67^. Same pattern observed in our study that ABA treatment increased spike numbers under drought conditions when compared with control.

Reduced water availability in the plant as a result of drought stress reduces the number of fertile tillers. When plants have less access to water, their metabolic activities slow down, resulting in fewer tillers^68^. The number of tillers is reduced by 14.6% during the tillering stage, 20.4% during the blooming stage, and 27.4% during the grain-filling stage when drought is applied. In the face of drought, ABA showed its greatest effectiveness in increasing the number of tillers. According to Sabagh et al.^69^, the capacity of ABA to enhance plant development is context-dependent.

The economic yield is very sensitive to the quantity of spikelets per spike. A decrease in spike length as a consequence of drought stress leads to a drop in spikelet density^61^. Drought during wheat grain-filling phase resulted in a decrease in spikelet production. The outcomes of ABA are equally noteworthy. Wheat plants with ABA_2_ application had the most spikelets per spike. He et al.^28^ found that plant hormones boost plant vegetative development by inducing the synthesis of chemicals that mitigate the negative effects of drought. According to research by Raza et al.^71^ grains per spike were significantly impacted by drought stress at any growth stage. The quantity of grains produced per spike shortens and the length of the spike under drought stress.

According to Tripathi et al.^72^, ABA may stimulate more robust plant development. Plant growth regulators boost proline production, which benefits plant health. In comparison to the control, seed priming with ABA_1_ and ABA_2_ increased grain yield. Zulfiqar et al.^10^ found that drought stress reduces grain weight, due to stunted development and reduced nutrient absorption by plants under water deficit. The molecule Indole-3-acetic acid is produced by ABA, and proline synthesis also aids in raising the drought threshold^73^. Plants that produce growth-regulating chemicals have better metabolic activities and a greater sink capacity, both of which contribute to a heavier 1000-grain yield. ABA_1_ and ABA_2_ in the current study had positively increase 1000-grain weight.

Plants under drought stress saw a drop in biological output at the tillering stage, flowering stage, and the grain-filling stage. Drought-related reductions in grain weight and plant height reduced biological yield^44,62^. ABA promotes optimal plant development^74^. ABA aids in enhancing nutrient absorption, which in turn imparts physiological changes in plant development, which in turn leads to a greater biological yield. According to Tripathi et al.^72^ research, the capacity of various ABA applications to stimulate plant development varies. Same trend was seen in this research for biological yield when ABA_1_ and ABA_2_ were applied.

Grain yield was reduced due to drought stress in every drought stages. At the anthesis stage, Zulfiqar et al.^10^ found that drought stress was most damaging to grain output. Water scarcity stress also reduces grain weight and yield by disrupting the absorption of nutrients. Under drought circumstances, the grain yield of ABA_1_ and ABA_2_ was raised as compared to the control treatment. These results have similar pattern with the previous studies.

As the temperature and humidity were both regulated, water use efficiency improved. When comparing tillering, blooming, and grain-filling drought, the control treatment had greater WUE. According to Lu et al.^75^ WUE improved when their interconnected units were used to their fullest extent. The use of ABA in wheat crops has been shown to increase both plant function and plant WUE. ABA_1_ and ABA_2_ foliar application, respectively, resulted higher WUE compared to the control when tested under drought circumstances which were matched with previous studies.

The chlorophyll level of a leaf is crucial to the plants ability to make nourishment. When leaf area is reduced, chlorophyll concentration is also reduced^76^. In addition, researchers^44,71^ found that drought stress reduced the plant's leaf area (LA), and a drop in LA resulted in a decrease in chlorophyll concentration. According to research by Raza et al.^60^ production of ROS that are harmful to chloroplasts increases in response to drought stress. ABA administration resulted in enhanced metabolic processes, decreased ROS levels, and increased cell proliferation. When ABA_1_ and ABA_2_ applied, the chlorophyll content increased, compared to the control in a drought experiment.

Furthermore, oxidative stress can detrimentally affect a plants biological activity through lipid peroxidation and nucleic acid damage^77,78^. It is essential to strike a balance between ROS generation and breakdown for optimal plant development^78^. For this reason, plants are vulnerable to oxidative stress since they cannot detoxify ROS^79,80^. Our findings revealed that during drought stress, plant EL, H_2_O_2_ concentration, and POD activity all increased, whereas SOD and CAT activities decreased. In addition, ABA role in maintaining and restoring wheat plant cell membranes contributed considerably to a reduction in ROS. According to Wang et al.^27^ when plants are subjected to biotic and abiotic stress, the electric transport chain becomes unbalanced, leading to an increase in the formation of ROS, which are harmful to cells. Following this, the plants immune system protects the cells by using anti-oxidant enzymes including SOD, CAT, and POD^81^.

These findings align with previous research indicating that olive cultivars with elevated levels of antioxidant enzymes have greater resistance to drought^82^ and same results for rapeseed^83^ brassica napus^84^. Furthermore, the heightened SOD activity seen after the application of ABA indicates the effective functioning of the antioxidant system inducer in protecting plants from oxidative damage. ABA enhances the activities of SOD, glutathione reductase, and catalase, hence reducing the detrimental effects of drought on plants^13,25,27^.

N, P, and K is the most important and abundant nutrient in plants, playing a vital role in growth and development^59^. The application of ABA significantly increased the N, P, and K contents in wheat grains under drought conditions^60^. This enhancement can be attributed to multiple mechanisms: ABA acts as a phytohormone that helps plants respond to drought by reducing water loss and promoting stress tolerance^85^. It also facilitates the uptake and transport of essential nutrients from the soil to the plant, particularly crucial when nutrient availability is limited^86^. Furthermore, Trapeznikov et al.^87^ stated that ABA promotes root development and nutrient remobilization, ensuring optimal nutrient levels even under stress.

Lastly, ABA induces the expression of genes related to stress tolerance and nutrient utilization as reported by Wei et al.^88^. In our research, major N-uptake was seen during the grain filling stage, while the lowest was shown under control conditions without drought stress. N absorption during the grain filling stage was greater than the control treatment. The highest values for P-uptake were observed when ABA_2_ was applied. The correlation between N uptake and K-uptake is consistent. Correlation analysis revealed that ABA effectively enhances SC, WUE, and chlorophyll content, and mitigates the adverse effects of drought stress.

## Conclusion

In the present study, water deficiency had a significant impact on both wheat growth and yield. However, the foliar application of ABA at different wheat growth stages was shown to be effective in improving wheat germination, growth, physiological, water-related characteristics, and yield, even under drought conditions. Specifically, we observed that drought stress during the grain-filling stage resulted in the greatest reduction in grain production. Importantly, we found that ABA application at a concentration of 200 mg/L was the most effective in mitigating the negative effects of drought in wheat. Our findings suggest that the use of ABA could be a valuable tool for enhancing wheat crop yields in dry agricultural systems. Further research is needed to evaluate the effectiveness of such applications under field conditions.

## Data Availability

All data generated or analyzed during this study are included in this published article.
